# Impact of rapid maxillary expansion on nasomaxillary complex volume in mouth-breathers

**DOI:** 10.1590/2177-6709.22.3.079-088.oar

**Published:** 2017

**Authors:** Mario Cappellette, Fabio Eduardo Maiello Monteiro Alves, Lucia Hatsue Yamamoto Nagai, Reginaldo Raimundo Fujita, Shirley Shizue Nagata Pignatari

**Affiliations:** 1 Universidade Federal de São Paulo (UNIFESP/EPM), Department of Otorhinolaryngology and Head and Neck Surgery, São Paulo, Brazil.

**Keywords:** Maxillary deficiency, Maxillary expansion, Computed tomography, Nasal cavity.

## Abstract

**Objective:**

To assess the volumetric changes that occur in the nasomaxillary complex of mouth-breathing patients with transverse maxillary deficiency subjected to rapid maxillary expansion (RME).

**Methods:**

This was a controlled, prospective intervention study involving 38 mouth-breathing patients presenting with transverse maxillary deficiency, regardless of malocclusion type or race. Twenty-three of them comprised the experimental group, which was composed of 11 (47.8%) boys, and 12 (52.2%) girls, with a mean age of 9.6 years, ranging from 6.4 to 14.2 years and standard deviation of 2.3 years; and 15 of them comprised the control group, composed of 9 (60%) boys and 6 (40%) girls with an mean age of 10.5 years, ranging from 8.0 to 13.6 years, and standard deviation of 1.9 years. All patients were scanned (CT) according to a standard protocol: Initial CT (T_1_), and CT three months thereafter (T_2_), and the patients in the experimental group were treated with RME using a Hyrax expander for the correction of maxillary deficiency during the T_1_-T_2_ interval. The CT scans were manipulated using Dolphin^®^ Imaging version 11.7 software for total and partial volumetric assessment of the nasomaxillary complex.

**Results:**

The results revealed that in the experimental group there was a significant increase in the size of the structures of interest compared to the control group, both in general aspect and in specific regions.

**Conclusions:**

Rapid maxillary expansion (RME) provided a significant expansion in all the structures of the nasomaxillary complex (nasal cavity, oropharynx, right and left maxillary sinuses).

## RESUMO

**Objetivo:**

avaliar as alterações volumétricas do complexo nasomaxilar em pacientes respiradores bucais com deficiência transversal da maxila tratados com expansão rápida da maxila (ERM).

**Métodos:**

foram selecionados 38 pacientes respiradores bucais, independentemente da má oclusão ou raça, portadores de constrição maxilar, e realizou-se um estudo de intervenção, prospectivo, controlado. Para o grupo experimental, foram selecionados 23 pacientes, sendo 11 (47,8%) meninos e 12 (52,2%) meninas, com média de idade de 9,6 anos (variando de 6,4 a 14,2 anos) e desvio-padrão de 2,3 anos; e 15 pacientes para o grupo controle, sendo 9 (60%) meninos e 6 (40%) meninas, com média de idade de 10,5 anos (variando de 8,0 a 13,6 anos) e desvio-padrão de 1,9 anos. Todos os pacientes realizaram exames de tomografia computadorizada (TC), de acordo com protocolo padronizado: TC inicial (T_1_) e TC após três meses do período de contenção (T_2_). Os pacientes do grupo experimental foram tratados com expansão rápida da maxila usando um expansor Hyrax para a correção da deficiência transversal durante o intervalo T_1_-T_2_. As imagens tomográficas para avaliação volumétrica total e parcial do complexo nasomaxilar foram manipuladas no programa *Dolphin* ^®^ versão 11.7.

**Resultados:**

os resultados revelaram que no grupo experimental houve um aumento significativo no tamanho das estruturas de interesse em comparação com o grupo controle, tanto no geral quanto nas regiões específicas.

**Conclusões:**

a expansão rápida da maxila (ERM) promoveu uma expansão significativa de todas as estruturas do complexo nasomaxilar (cavidade nasal, orofaringe, seios maxilares direito e esquerdo).

## INTRODUCTION

Transverse maxillary deficiency associated with respiratory problems is a condition frequently observed in otorhinolaryngology (ENT) and orthodontic practice. This type of malocclusion warrants special attention by orthodontists, otolaryngologists and allergists since its causes and symptoms are clearly related to these three specialties. Besides, the transverse maxillary deficiency can be treated by means of rapid maxillary expansion (RME), thus improving the nasal airflow and the breathing pattern.^1^

In 1860, Angell first described a method, known as maxillary expansion, for treating patients with generalized lack of space in the maxillary arch, and transverse maxillary deficiency. Eysel was the first rhinologist to study the effects of maxillary expansion on nasal cavity dimensions in the year 1886, and noted that in the period following maxillary expansion several changes occurred in the maxilla such as increased nasal width near the midpalatal suture.^1^ ^,^ ^2^

Later, other studies showed that histological repair of the connective tissue occurs in the midpalatal suture during and after the active expansion phase, as well as changes in the anatomy of the septum and nasal cavity, triangular opening of the midpalatal suture, with the apex facing the nasal cavity, and improved nasal breathing.^3^ ^-^ ^7^

Since then, numerous articles in the scientific literature have reported the benefits of rapid maxillary expansion for the nasal cavity, also confirmed in ear, nose and throat (ENT) practice. These studies used posteroanterior radiography (PA), thus complementing the evaluation of transverse alterations, as well as cephalometrics, acoustic rhinometry, and computed tomography - with or without the concurrent use of imaging software^5^ ^-^ ^16^-, demonstrating significant increase in the cross-sectional dimensions of the nasal cavity, volumetric increase and reduction in nasal resistance. The enlargement of nasal cavity with an increase of nasal volume could diminish the resistance of nasal airflow and improve a nasal breathing. However, these effects depend on the existence or not of nasal obstruction and on its location and severity. Patients with nasal obstructions such as turbinate hypertrophy or septum deviations were excluded from the study.

Several methods and imaging software have been used to confirm the expansion of the nasomaxillary complex and its adjacent structures after RME.^16^ ^,^ ^17^

Imaging software programs have been extremely useful in helping to assess the benefits of RME. They have also proven vital for structural comparisons between pre and post-clinical treatment, and to evaluate the morphological changes caused by the treatment, since they improve the visualization of anatomical structures by rendering unnecessary the superimposition of conventional radiographs. Furthermore, these programs enhance the accuracy of research findings, besides improving the effectiveness of any techniques applied, while facilitating the use of computer tools for 3D image manipulation, either by itself or associated with other software.^6^

The purpose of this study was to investigate the impact of rapid maxillary expansion (RME) on the volume of the nasomaxillary complex, using computed tomography (CT) associated with an image manipulation software.

## MATERIAL AND METHODS

This was a controlled, prospective intervention study involving 38 mouth-breathing patients presenting with transverse maxillary deficiency, regardless of malocclusion type or race. The experimental group consisted of 23 patients (11 female and 12 male) with a mean age of 9.6 years ranging from 6.4 to 14.2 years. Fifteen patients were selected for the control group (9 male and 6 female), with a mean age of 10.5, ranging from 8.0 to 13.6 years. All patients were in mixed or permanent dentition, with a diagnosis of mouth-breathing and maxillary deficiency. 

The following diagnostic exams were applied: 1) standardized questionnaire originally designed to measure the quality of life of patients with sleep breathing disorders after adenotonsillectomy - which comprised six domains concerning physical suffering, sleep disturbance, speech or swallowing problems, emotional distress, activity limitation, and degree of parents/legal guardians’ concern about their own child’s snoring -; 2) ENT evaluation that verified the presence of nasal obstruction after anterior rhinoscopy, oroscopy and nasofiberendoscopy in order to check for mouth-breathing pattern; 3) orthodontic evaluation that observed the narrowing of the upper arch, with a ogival palate. For their breathing assessment, some clinical tests such as the steam breath against a mirror and the water remains in the patient’s mouth with the lips closed for 3 minutes were performed.

Syndromic patients or patients with craniofacial abnormalities such as Pierre-Robin and Treacher-Collins, among others, potential candidates for adenoidectomy or adenotonsillectomy, septum deviation, complete obstruction of the nasal cavity by nasal turbinates, anatomic alterations of the nasal septum, intranasal tumors or polyps, adenoid occupying more than 70% of the choanas, purulent secretions in the middle nasal meatus or in the floor of the nose, and patients with dental or periodontal changes were excluded from the study. This study was approved by the Committee for Ethics in Institutional Research of the Federal University of São Paulo (registered under #164761).

All CT scans were performed in the Department of Diagnostic Imaging of the institution, using a multislice device (Philips^®^ Brilliance CT scanner 64 channels).

All tests confirmed the presence of maxillary deficiency, and all patients were subjected to the same tomographic evaluation protocol, T_1_ (CT1), at baseline, and T_2_ (CT2), about 3 months after the first CT scan. The patients of experimental group treatment were treated following the same protocol: Hyrax expander was attached to the maxillary second primary molars and extended forward to the palatal surfaces of the primary canines (2-banded) or supported by bilateral maxillary first premolars and first molars (4-banded). After insertion, the six initial activations of the appliance were applied by the orthodontist. Subsequent activations were performed by the legal guardians, who were instructed to make two daily activations, with no interval between them. This procedure went on until RME was achieved, within a period ranging from 15 to 20 days. After this period the appliance was kept in place for nearly 3 months, and removed after bone formation was observed through occlusal radiographs. In this phase, parents/legal guardians answered the same quality-of-life questionnaire. Thereafter, patients were immediately subjected to a new CT scan (CT2).

Volumetric measurements and comparisons between images of both groups, CT1 and CT2, were carried out with the aid of Dolphin^®^ Imaging v. 11.7 software, using the “Airway Volume” tool, and density was set at 65 for all patients. Volumetric measurements and comparisons between images of both groups were carried out using the “Airway Volume” tool, which works filling the structures according to theirs density or Hounsfield units (0-100).

**Figure 1:**
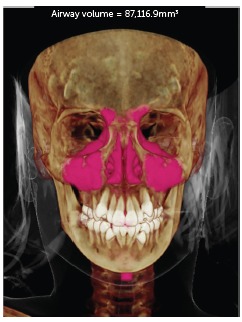
Total initial volume.

**Figure 2:**
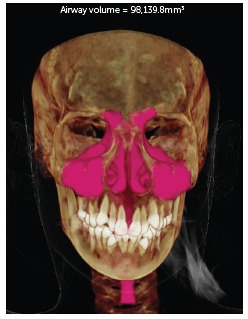
Total final volume.

The images were evaluated in three views (sagittal, coronal and axial), thus delimiting the nasomaxillary complex, and then calculating the volume in cubic millimeters. The results were statistically analyzed and compared as shown in Figures 1-2.

The total of 38 patients as a sample size was considered statistically appropriate. The Table 2 shows the volumetric evaluation of the nasomaxillary complex at T_1_ and T_2_. Normality of distribution of increases in total volume, nasal volume, oropharynx and right and left maxillary sinuses was assessed with the Shapiro-Wilk test (Table 3). Tables 4 and 5 show the statistical power of the sample in all comparison of interest.

**Table 1:** Distribution of sex and age of children in the control and experimental groups.

		control (n=15)		experimental (n=23)		Total (n=38)		P
Sex	male	9	60.0%	11	47.8%	20	52.60%	0.463^a^
	female	6	40.0%	12	52.2%	18	47.40%	
	Total	15	100.0%	23	100.0%	38	100.00%	
Age (years)	Mean	10.5		9.6		10.0		0.204^b^
	median	10		9.5		9.8		
	minimum	8		6.4		6.4		
	maximum	13.6		14.2		14.2		
	standard deviation	1.9		2.3		2.2		

**Table 2:** Summary-measurements (mm^3^) of total (TV) and nasal (NV) volumes, oropharynx (Oro); right (RMS) and left (LMS) maxillary sinuses, of children in the control and experimental groups

	control (n=15)					experimental (n=23)				
	mean	median	minimum	maximum	SD	mean	median	minimum	maximum	SD
TV (T_1_)	55,567.8	52,378.3	47,554.4	68,785.7	8,104.9	59,823.4	59,667.3	42,222.6	87,116.9	11,502.8
TV (T_2_)	55,757.2	52,878.6	47,575.3	68,889.6	8,239.5	69,322.4	67,821.3	51,513.7	98,139.8	11,867.5
TV increase*	189.4	500.3	20.9	103.9	134.6	9,499	8,154	9,291.1	11,022.9	364.7
NV (T_1_)	34,426	33,108.3	29,207.4	43,479.4	5,059	33,418.7	31,587.2	25,985.2	50,792.1	6,107.6
NV (T_2_)	34,488.7	33,200.4	29,214.9	43,487.3	5,088.9	38,450.6	37,853.7	30,271.9	58,035.6	6,329.1
NV increase*	62.7	92.1	7.5	7.9	29.9	5,031.9	6,266.5	4,286.7	7,243.5	221.5
Oro (T_1_)	7,531	7,215.9	5,682.9	10,784.7	1,535	10,262.3	9,748.1	6,760.5	14,449.6	2,421.1
Oro (T_2_)	7,572.4	7,300.7	5,693	10,802.2	1,526.4	12,955.1	13,584.1	8,295	18,345.6	2,942.8
Oro increase*	41.4	84.8	10.1	17.5	-8.6	2,692.8	3,836	1,534.5	3,896	521.7
RMS (T_1_)	8,795.9	8,149.1	7,105.7	11,268.3	1,594.2	9,161.9	8,813.9	3,068.3	16,942.4	2,836.7
RMS (T_2_)	8,831.4	8,149.3	7,162.7	11,273.3	1,582.6	11,343.2	11,668.8	5,746.2	17,968.4	2,807.7
RMS increase*	35.5	0.2	57	5	-11.6	2,181.3	2,854.9	2,677.9	1,026	-29
LMS (T_1_)	8,763.8	8,456.1	6,226	11,105	1,580.4	8,999.8	8,260.7	4,392.8	17,689.7	3,046.1
LMS (T_2_)	8,799.8	8,487	6,228.2	11,187.1	1,603.3	11,371.2	11,505.4	4,823.9	19,639.5	3,140.4
LMS increase*	36	30.9	2.2	82.1	22.9	2,371.4	3,244.7	431.1	1,949.8	94.3

**Table 3:** Results of normality tests to measure increases in total volume, nasal volume, oropharynx, and right and left maxillary sinuses with the aim of determining the appropriate statistical test to compare the groups.

	control	Experimental
total volume increase	<0.001	0.173
nasal volume increase	<0.001	0.234
oropharyngeal increase	0.092	0.340
right maxillary sinus increase	0.093	0.385
left maxillary sinus increase	0.035	0.364

**Table 4:** Estimates of the sample power in comparison of the T_1_ and T_2_.

	Group	sample power in comparison of the T_1_ and T_2_
total volume	control experimental	> 0.9999
		0.7582
nasal volume	control experimental	> 0.9999
		0.5216
oropharyngeal	control experimental	> 0.9999
		0.6359
right maxillary sinus	control experimental	> 0.9999
		0.7048
left maxillary sinus	control experimental	> 0.9999
		0.7606

**Table 5:** Estimates of the sample power in comparison of the increases between experimental group and control group.

	Statistical power in comparison of the groups experimental and control
total volume	> 0.9999
nasal volume	0.9957
oropharyngeal	0.9024
right maxillary sinus	0.998
left maxillary sinus	0.9772

To estimate evaluator reliability and reproducibility, 10 randomly selected records were reevaluated after a month of preliminary data collection. All parameters were measured by the same evaluator. Normality was assessed with the Shapiro-Wilk test (*p*> 0.05). After that, paired sample *t*-tests were used to investigate the difference of both measurements and intraclass correlation coefficient (ICC) was used to test the intra-rater reliability.

The statistical treatment of the data was performed with the Statistical Package for the Social Sciences (SPSS), version 22 for Windows (Table 6).

**Table 6:** Normality of data: *p*-values of the Shapiro-Wilk test (n = 10).

Variables	T_1_		T_2_	
	Measurement 1	Repeat	Measurement 1	Repeat
Total volume	0.334	0.334	0.488	0.488
Nasal volume	0.312	0.312	0.448	0.448
Oropharyngeal	0.163	0.163	0.180	0.180
Right maxillary sinus	0.218	0.218	0.768	0.768
Left maxillary sinus	0.513	0.513	0.642	0.642

## NORMALITY OF DATA

Considering a significance level of 5%, there were no significant deviations from the normality of the data (*p*> 0.05), both in T_1_ and T_2_. For this reason, parametric tests were used to analyze the error and reliability of the measurements: Student’s *t*-test for paired samples and Intraclass Correlation Coefficient (ICC).

The results presented in Table 7 show a total correspondence between the initial measurements and the repetitions by the same evaluator (Intraclass correlation) in both T_1_ and T_2_. In fact, the means of the initial measurements and the repetitions were equal (p = 1,000) and the ICC equals 1,000 in all variables, indicating the absence of measurement error, and reliability and reproducibility.

**Table 7:** Error analysis: mean and standard deviation, Student’s t-test for paired samples and ICC (n=10).

Variables	Measurement 1	Repeat	Student’s t-test	ICC
	Mean + SD	Mean + SD		
T_1_				
Total Volume	61,398.2 (13,439.9)	61,398.2 (13,439.9)	1.000	1.000
Nasal volume	31,455.3 (4,169.9)	31,455.3 (4,169.9)	1.000	1.000
Oropharyngeal	9,773.6 (2,823.0)	9,773.6 (2,823.0)	1.000	1.000
Right maxillary sinus	10,001.7 (3,025.1)	10,001.7 (3,025.1)	1.000	1.000
Left maxillary sinus	9,968.0 (3,782.8)	9,968.0 (3,782.8)	1.000	1.000
T_2_				
Total Volume	68,987.5 (13,330.1)	68,987.5 (13,330.1)	1.000	1.000
Nasal volume	35,417.4 (3,662.4)	35,417.4 (3,662.4)	1.000	1.000
Oropharyngeal	11,905.1 (2,788.8)	11,905.1 (2,788.8)	1.000	1.000
Right maxillary sinus	11,624.2 (3,170.8)	11,624.2 (3,170.8)	1.000	1.000
Left maxillary sinus	11,344.8 (3,839.3)	11,344.8 (3,839.3)	1.000	1.000

Reliability and reproducibility results showed no error for volume variable, which can be attributed to analysis by specific tool of the software using a grey scale and automatic volume determination (Table 7).

## STATISTICAL ANALYSIS

Statistical analysis of all data collected in this research was initially performed descriptively using mean, median, minimum and maximum values, standard deviation, absolute and relative frequencies (percentage), in addition to individual profile graphs (line graph) and one-dimensional dispersion graphs. The inferential analysis employed in order to confirm or refute evidence found in the descriptive analysis comprised:

» Pearson’s Chi-square test,^28^ to compare the control and experimental groups with respect to gender.

» Student’s *t*-test for independent samples,^21^ to compare the control and experimental groups with respect to age (years), oropharyngeal expansion (mm^3^), and right maxillary sinus (mm^3^).

» Mann-Whitney test,^22^ to compare the control and experimental groups with respect to increases in total volume (mm^3^), nasal volume (mm^3^), and left maxillary sinus (mm^3^).

» Shapiro-Wilk test, ^29^ to evaluate normality in the distribution of increases in total volume (mm^3^), nasal volume (mm^3^), oropharynx (mm^3^), right (mm^3^) and left (mm^3^) maxillary sinuses, in the control *vs.* experimental groups.

A 5% significance level was applied to all results achieved through inferential analysis.

According to the power of the sample as shown in the Table 4, the sample was considered sufficient to verify the statistical differences between T_1_ and T_2_ both for experimental group and control group.

The comparisons of the increment between experimental group and control group showed significant sample power (Table 5).

## RESULTS

Demographic data of the selected sample are shown in Table 1.

The inferential results confirmed that both the control and experimental groups showed the same profile with respect to gender (*p*= 0.463), and age (*p*= 0.204).

**Figure 3:**
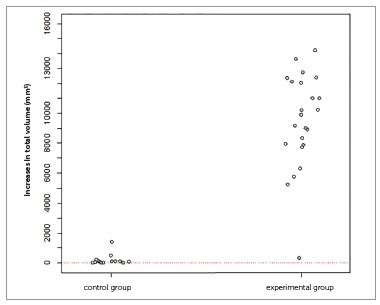
One-dimensional dispersion diagram of increases in total volume (mm^3^) of children in the control and experimental groups.

The volumetric evaluation of the nasomaxillary complex of 38 children at T_1_ and T_2_ can be seen in Table 2, and Figures 1 and 3. The black lines in Figures 1 and 2 represent the time evolution of each child. The red lines in those graphs represent the mean and standard error.

At T_1_, the control group exhibited a mean total volume of 55,567.8 mm^3^, ranging from 47,554.4 to 68,785.7 mm^3^, with a standard deviation of 8,104.9 mm^3^. The mean nasal volume was 34,426.0 mm^3^, ranging from 29,207.4 to 43,479.4 mm^3^, with a standard deviation of 5,059.0 mm^3^. Oropharynx mean was 7,531.0 mm^3^, ranging from 5,682.9 to 10,784.7 mm^3^, with a standard deviation of 1,535.0 mm^3^. The mean right maxillary sinus was 8,795.9 mm^3^, ranging from 7,105.7 to 11,268.3 mm^3^, with a standard deviation of 1,594.2 mm^3^. The mean left maxillary sinus was 8,795.9 mm^3^, ranging from 6,226.0 to 11,105.0 mm^3^, with a standard deviation of 1,580.4 mm^3^.

At T_2_, the control group showed a mean total volume of 55,757.2 mm^3^, ranging from 47,575.3 to 68,889.6 mm^3^, with a standard deviation of 8,239.5 mm^3^.

The mean nasal volume was 34,488.7 mm^3^, ranging from 29,214.9 to 43,487.3 mm^3^, with a standard deviation of 5,088.9 mm^3^. The mean oropharynx was 7,572.4 mm^3^, ranging from 5,693.0 to 10,802.2 mm^3^, with a standard deviation of 1,526.4 mm^3^. The mean right maxillary sinus was 8,831.4 mm^3^, ranging from 7,162.7 to 11,273.3 mm^3^, with a standard deviation of 1,582.6 mm^3^. On the left maxillary sinus, the mean was 8,799.8 mm^3^, ranging from 6,228.2 to 11,187.1 mm^3^, with a standard deviation of 1,603.3 mm^3^.

At T_1_, experimental group had a mean total volume of 59,823.4 mm^3^, ranging between 42,222.6 and 87,116.9 mm^3^ (SD = 11,502.8 mm^3^). The mean nasal volume was 33,418.7 mm^3^, ranging from 25,985.2 to 50,792.1 mm^3^, with a standard deviation of 6,107.6 mm^3^. The mean oropharynx was 10,262.3 mm^3^, ranging from 6,760.5 to 14,449.6 mm^3^, with a standard deviation of 2,421.1 mm^3^. The mean right maxillary sinus was 9,161.9 mm^3^, ranging from 3,068.3 to 16,942.4 mm^3^, with a standard deviation of 2,836.7 mm^3^. The left maxillary sinus had a mean of 8,999.8 mm^3^, ranging from 4,392.8 to 17,689.7 mm^3^, with a standard deviation of 3,046.1 mm^3^.

At T_2_, the experimental group had a mean total volume of 69,322.4 mm^3^, ranging from 51,513.7 to 98,139.8 mm^3^, with a standard deviation of 11,867.5 mm^3^. The mean nasal volume was 38,450.6 mm^3^, ranging from 30,271.9 to 58,035.6 mm^3^, with a standard deviation of 6,329.1 mm^3^. The mean oropharynx was 12,955.1 mm^3^, ranging from 8,295.0 to 18,345.6 mm^3^, with a standard deviation of 2,942.8 mm^3^. The right maxillary sinus was 11,343.2 mm^3^, ranging from 5,746.2 to 17,968.4 mm^3^, with a standard deviation of 2,807.7 mm^3^. The mean left maxillary sinus was 11,371.2 mm^3^, ranging from 4,823.9 to 19,639.5 mm^3^, with a standard deviation of 3,140.4 mm^3^.

In Figures 1 and 2, example of a treated patient, comparing pre- and post-treatment total volumes. As depicted in these Figures, all children experienced increases in total volume. It is noteworthy however that these increases were more significant in the experimental group than in the control group.

**Figure 4:**
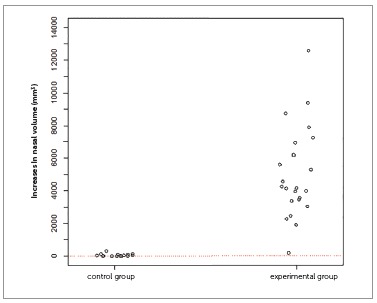
One-dimensional dispersion diagram of increases in nasal volume (mm^3^) of children in the control and experimental groups.

**Figure 5:**
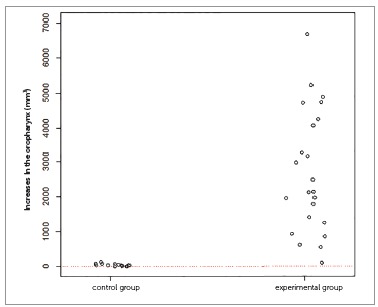
One-dimensional dispersion diagram of increases in the oropharynx (mm^3^) of children in the control and experimental groups.

**Figure 6:**
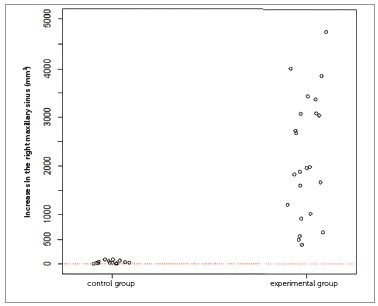
One-dimensional dispersion diagram of increases in the right maxillary sinus (mm^3^) of children in the control and experimental groups.

**Figure 7:**
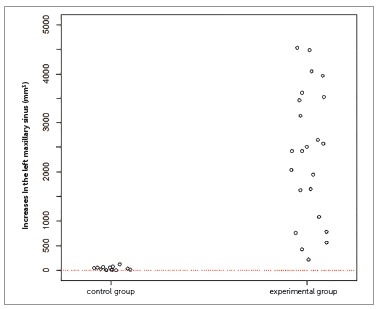
One-dimensional dispersion diagram of increases in the left maxillary sinus (mm^3^) of children in the control and experimental groups.

The inferential results (Figs 3 to 7) confirmed the evidence obtained in the descriptive analyses, meaning that the increases in total volume (*p*< 0.001), nasal volume (*p*< 0.001), oropharynx (*p*< 0.001), and right (*p*< 0.001) and left (*p*< 0.001) maxillary sinuses in the experimental group were more statistically significant than in the control group.

## DISCUSSION

Transverse maxillary deficiency associated with respiratory problems has been widely discussed by orthodontists and otolaryngologists, given the relationship between causes, effects and treatment. Today, rapid maxillary expansion is regarded as an important method to correct maxillary deficiency. Since it was first introduced in 1860 in the United States, Angell’s technique has been validated by many other authors as it makes possible the splitting of the midpalatal suture while producing certain changes in the nasal cavity, which improve breathing.^1^ ^,^ ^3^ ^-^ ^8^ ^,^ ^11^ ^,^ ^14^ ^,^ ^17^

Regarding diagnosis and treatment planning most scientific studies which analyzed the effectiveness of the rapid maxillary expansion therapy used posteroanterior (PA) radiographs as an evaluation method. Radiographs enable an analysis of the transverse dimensions of the face by providing a broader view for the diagnosis of crossbites and orthopedic changes, i.e., this is a technique that provides reliability when comparing skeletal cephalometric points to dental cephalometric points.^10^ Unfortunately, the superimposition of radiographs of the anatomical structures can compromise the accuracy with which these points are marked.

With the development of imaging tests in the 1970s, computed tomography (CT) has been increasingly used to ensure reliable images, and has by now garnered a reputation as a new parameter for many health care areas since craniometrics points can be found with greater precision, unlike posteroanterior radiographs, which can be distorted. CT has rendered research results more accurate besides improving techniques such as the use of computer tools for 3D image manipulation.^8^ ^,^ ^17^

Increases in nasal width and height were observed by posteroanterior radiographs and/or CT at different stages of RME by several authors, who corroborated the results achieved in this study. According to the literature, changes in nasal volume between pre and post-RME assessed by computed tomography have been observed by several authors. This was also among the goals of this study, which were confirmed by the results.^1^ ^,^ ^3^ ^,^ ^5^ ^-^ ^8^ ^,^ ^10^ ^,^ ^11^ ^,^ ^16^ ^,^ ^17^ ^,^ ^20^

No doubt that an improved breathing pattern is an important clinical achievement, as observed in this study immediately following RME, and as reported by patients and legal guardians alike, although this was not the aim of this study. As disclosed is published studies, our patients also showed an increase in nasal cavity volume after RME, with this outcome being confirmed by an image manipulation program with 3D images, and by quantification of the measured areas.^3^ ^,^ ^6^ ^-^ ^8^ ^,^ ^11^ ^,^ ^17^ The same results were observed in all measures of the nasomaxillary complex,^8^ ^,^ ^16^ ^,^ ^17^ despite differences in the maxillary expansion protocols of the various studies.

Some studies have failed to show gains in some nasomaxillary complex structures, particularly in the volume of the maxillary sinuses^18^ and nasal cavity.^19^ However, increases in oropharyngeal volume have been reported.^20^

The RME produced significant width increases in the maxilla and nasal cavity which are important for stability of the treatment improving respiratory function and craniofacial development. De Felippe et al,^23^ by means of 3D morphometric analysis and of acoustic rhinometry evaluation, found an increase in the area of the nasal cavity, concomitant with a reduction in nasal airway resistance immediately after RME. These authors also observed stability of the results in a long-term follow-up (60 months after RME), with values comparable to those of subjects with normal nasal breathing conditions.

The breathing stage of the patients is difficult to control which has influence on the airway size. Thereby, patients with any obstruction of the nasal cavity or anatomic alterations of the nasal septum were excluded from the study. The examination of the upper airway plays an important role in the evaluation of the growth and general health of subjects with breathing disorders.^24^ ^,^ ^25^ Despite reduction in resistance after RME, only a few attempts have been made to investigate whether such changes are capable of causing significant improvements on respiration, physical activities and quality of life of mouth-breathers.^26^

The analysis of results of the questionnaire obtained after RME suggests that the severity of the respiratory symptoms reduced after RME. Iwasaki et al.^27^ related that the changes after RME, as measured by objective tests of nasal airway patency such as rhinomanometry and acoustic rhinometry, show improved conditions for nasal breathing up to 11 months after RME.

According to the results of this study showing increased intranasal capacity and considering other studies^7^ ^,^ ^10^ reporting the impact of RME on the quality of the life of mouth-breathers with improvement of the breathing pattern, the RME may favor the nasal function, which is an important factor in these growing patients.

## CONCLUSIONS

The results showed that rapid maxillary expansion (RME) induces a volumetric expansion in the nasomaxillary complex as well as in all its structures, the nasal cavity, oropharynx and maxillary sinuses, individually.
